# Impaired 24-h activity patterns are associated with an increased risk of Alzheimer’s disease, Parkinson’s disease, and cognitive decline

**DOI:** 10.1186/s13195-024-01411-0

**Published:** 2024-02-14

**Authors:** Joseph R. Winer, Renske Lok, Lara Weed, Zihuai He, Kathleen L. Poston, Elizabeth C. Mormino, Jamie M. Zeitzer

**Affiliations:** 1 Department of Neurology and Neurological Sciences, Stanford University School of Medicine, 453 Quarry Road, Palo Alto, CA 94304 USA; 2 Department of Psychiatry and Behavioral Sciences, Stanford University, Stanford, CA USA; 3 Department of Bioengineering, Stanford University, Stanford, CA USA; 4 Wu Tsai Neurosciences Institute, Stanford University, Stanford, CA USA; 5 Sierra-Pacific Mental Illness Research, Education, and Clinical Center (MIRECC), Veterans Affairs Palo Alto Health Care System, Palo Alto, CA USA

**Keywords:** Rest-activity rhythms, Alzheimer’s disease, Parkinson’s disease, Cognitive aging

## Abstract

**Background:**

Sleep-wake regulating circuits are affected during prodromal stages in the pathological progression of both Alzheimer’s disease (AD) and Parkinson’s disease (PD), and this disturbance can be measured passively using wearable devices. Our objective was to determine whether accelerometer-based measures of 24-h activity are associated with subsequent development of AD, PD, and cognitive decline.

**Methods:**

This study obtained UK Biobank data from 82,829 individuals with wrist-worn accelerometer data aged 40 to 79 years with a mean (± SD) follow-up of 6.8 (± 0.9) years. Outcomes were accelerometer-derived measures of 24-h activity (derived by cosinor, nonparametric, and functional principal component methods), incident AD and PD diagnosis (obtained through hospitalization or primary care records), and prospective longitudinal cognitive testing.

**Results:**

One hundred eighty-seven individuals progressed to AD and 265 to PD. Interdaily stability (a measure of regularity, hazard ratio [HR] per SD increase 1.25, 95% confidence interval [CI] 1.05–1.48), diurnal amplitude (HR 0.79, CI 0.65–0.96), mesor (mean activity; HR 0.77, CI 0.59–0.998), and activity during most active 10 h (HR 0.75, CI 0.61–0.94), were associated with risk of AD. Diurnal amplitude (HR 0.28, CI 0.23–0.34), mesor (HR 0.13, CI 0.10–0.16), activity during least active 5 h (HR 0.24, CI 0.08–0.69), and activity during most active 10 h (HR 0.20, CI 0.16–0.25) were associated with risk of PD. Several measures were additionally predictive of longitudinal cognitive test performance.

**Conclusions:**

In this community-based longitudinal study, accelerometer-derived metrics were associated with elevated risk of AD, PD, and accelerated cognitive decline. These findings suggest 24-h rhythm integrity, as measured by affordable, non-invasive wearable devices, may serve as a scalable early marker of neurodegenerative disease.

## Background

Alzheimer’s disease (AD) and Parkinson’s disease (PD) are the two most prevalent neurodegenerative diseases, with numbers predicted to increase in the coming decade [1, 2]. Common to both diseases is a prodromal phase during which misfolded proteins begin to spread throughout the brain, often years before clinical diagnosis [3, 4]. Altered sleep and circadian rhythms, thought to result from the earliest pathology within brainstem nuclei, frequently manifest as one of the initial symptoms during this prodromal disease stage in both AD and PD [5–7]. Thus, detection of disrupted 24-h patterns of activity has the potential to serve as an early marker for identifying both AD and PD.

Supporting this hypothesis, previous work using wrist-worn accelerometers has demonstrated that specific impairments in 24-h activity patterns are associated with accelerated cognitive decline [8–12], an increased risk of developing AD [8, 9, 13, 14], and in one study, an increased risk of developing PD [15]. As more older adults elect to track their daily physical activity and sleep using consumer-grade devices, attention has turned to how these patterns of activity can be leveraged in large, community-based samples. However, few studies have utilized objective assessments of 24-h activity in large cohorts of older adults, and those that have are limited by sample sizes under 5000 with limited numbers of individuals converting to AD or PD during the follow-up period [9, 12, 13, 15, 16]. The UK Biobank dataset, a large community-based sample of middle-aged and older adults in which more than 80,000 participants had week-long activity recordings, provides a unique opportunity to explore these associations in an unprecedently large sample of individuals.

We determined whether accelerometer-derived metrics of 24-h activity patterns are associated with longitudinal cognitive test performance and risk of incident AD and PD in the UK Biobank cohort. 24-h accelerometer data can be quantified via several complementary methods to capture different information about an individual’s sleep-wake rhythmicity and activity patterns. By utilizing three approaches, we sought to characterize integrity of sleep-wake rhythmicity through (1) the robustness of activity patterns (with cosinor methods), (2) the fragmentation and regularity of 24-h rhythms (with nonparametric methods), and (3) data-driven patterns to detect differences in individuals who developed AD and PD (functional principal component analysis). We hypothesized that greater fragmentation of sleep-wake rhythmicity and lower levels of activity would be prospectively associated with increased risk of AD, PD, and declining cognitive performance.

## Methods

Wrist-worn triaxial accelerometry [AX3, Axivity, Newcastle upon Tyne, UK] was obtained over 1 week from a large community-based sample of adults living in England, Wales, and Scotland participating in the UK Biobank study, an ongoing community-based cohort study. More than 500,000 participants aged between 40 and 69 years were recruited in 2006–2010 (full details available in Doherty et al. 2017 [17]). The UK Biobank study did not exclude based on any health condition; age was the only eligibility criteria for participating in the study. No restrictions were placed on behavior during the week of accelerometer data collection. Data were downloaded March 2022.

### Participants

Accelerometer data (UK Biobank data field 90001) were collected from June 2013 to January 2016 and included 103,671 individuals. Of these, 5 withdrew from the study, 4687 had accelerometer data marked as unreliable (90002), and 1 had unreliable calibration data (90016). An additional 7567 individuals were excluded due to wearing the accelerometer for less than 5 days (90051) and 7479 were excluded because their data were collected during a daylight savings time crossover (90018) or the week following a daylight savings time shift. Data from the remaining 83,932 individuals were extracted from the UK Biobank and processed. Of these, an additional 1097 were excluded due to having at least one off-wrist period 24-h or longer, and 6 were excluded due to having implausibly high acceleration values (> 2000). The remaining accelerometer data sample comprised 82,829 individuals.

### Accelerometer data

Accelerometer data collection in the UK Biobank did not include concurrent sleep logs or sleep timing information. Our analyses characterize 24-h sleep-wake rhythms rather than purporting to detect sleep based on activity alone.

#### Preprocessing

**Fig. 1:**
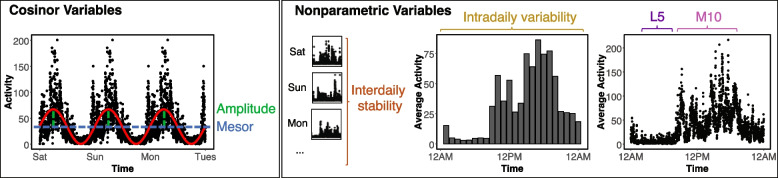
Quantitative metrics of 24-h activity patterns. Left, cosinor variables are calculated by fitting a cosine function to the accelerometer data. Mesor (midline-estimating statistic of rhythm) is the mean activity level of the cosine wave. Amplitude is defined as the distance between the mean activity level and the peak of the cosine wave. Right, nonparametric variables do not require fitting a shape to the accelerometer data. Interdaily stability is defined as the regularity of the 24-h rhythm across days, whereas intradaily variability represents the fragmentation of activity within the average 24-h day. L5 (least active 5 h) represents the mean activity level during what is typically time in bed, and M10 (most active 10 h) represents mean activity level during the day

High-frequency (100 Hz) accelerometer data were processed on Sherlock, a high-performance computing cluster provided by Stanford University, using the steps outlined in Weed et al. 2022 [18]. In brief, data spanning 1 week of collection were down-sampled to 30 s epochs using the biobankAccelerometerAnalysis package in Python v3.6.1 [17]. Non-wear time was defined as stationary episodes lasting for at least 60 min in which all three axes had a standard deviation of less than 13.0 mg. If present, non-wear segments were automatically imputed using the median of similar time-of-day vector magnitude and intensity distribution data points with 30-s granularity on different days of the measurement [18]. Following these preprocessing steps, we derived the following six metrics. Figure 1 shows accelerometer data from a representative individual to illustrate each of the six metrics.

#### Cosinor analyses

Cosinor analyses (fitting a cosine wave to the data) were performed using the cosinor2 package in R [19] and resulted in two metrics of interest: (1) mesor (rhythm-adjusted mean activity) and (2) amplitude (half the difference between peak and nadir of fitted cosine wave). Mesor and amplitude reflect activity levels and thus have no upper limit.

#### Nonparametric analyses

Nonparametric analyses were conducted with nparACT package in R [20, 21] to derive four metrics: (1) intradaily variability (IV; fragmentation of activity within 24-h periods), (2) interdaily stability (IS; regularity of activity across 24-h periods), (3) activity level during the least active 5 h (L5), and (4) activity level during the most active 10 h (M10). IV values can vary from 0 to 2, with lower values representing greater consolidation of activity within days and higher values indicating greater fragmentation. IS values can vary from 0 to 1, with higher values indicating greater regularity across days. L5 and M10 reflect activity levels and thus have no upper limit.

#### fPCA

Functional principal component analyses (fPCA) were performed using the fpca package in R [22]. Following previously established methods [22–24], each individual’s 24-h median accelerometer data were fit with a nine-Fourier-based function. These functions were examined with functional data analysis to determine orthogonal components that explained the most variance across individuals. The first four fPCA components (explaining > 90% of the variance) were used for analysis, with individuals in a sample scored on the magnitude of each component contributing to their activity pattern. These scores were extracted and compared between disease progressors and non-progressors. As the results of fPCA are dependent on the specific subsets of data analyzed, we conducted two separate fPCA analyses, consisting of individuals who progressed to AD or PD (see below) and matched controls who did not progress to AD or PD (1:1 match on age at accelerometer collection, sex, education, general health, body mass index, and Townsend deprivation index, using the MatchIt package [25] in R).

### Ascertainment of incident AD and PD

Incident AD or PD was ascertained from algorithmically defined UK Biobank variables that utilized a combination of self-report and ICD-10 codes from medical records to determine the first recorded date of a diagnosis (AD, data field 42020; PD, 42032) [26–28]. An individual would be considered to have developed AD or PD during the study period if they had an initial diagnosis of AD or PD between the time of actigraphy data collection (as early as June 2013) and September 2021. Individuals with AD (*n* = 13) and PD (*n* = 133) diagnoses preceding their actigraphy data collection were excluded from the analyses.

### Longitudinal cognitive data

Cognitive tests were designed specifically for the UK Biobank and were administered unsupervised using a computer touchscreen interface either during an in-person visit or remotely [29]. A subset of individuals with accelerometer data completed cognitive testing. Individuals were included in the analysis for a given test if they had (1) a test score within a year of their accelerometer data collection and (2) at least one follow-up test score. Five test scores were included from the UK Biobank cognitive battery: Trail Making Test A (numeric; 20156 and 6348; *n* = 6,731; 4.3 ± 1.1 years of follow-up) and Trail Making Test B-A (alphanumeric-numeric; 20157 and 6350; *n* = 6,629; 4.3 ± 1.1 years of follow-up), Symbol Digit Substitution Test (23323, 23324, 20159, and 20195; *n* = 7,533; 4.3 ± 1.1 years of follow-up), Numeric Memory Test (4282 and 20240; *n* = 7,401; 4.3 ± 1.1 years of follow-up), and Fluid Intelligence Test (20016 and 20191; *n* = 11,030, 3.3 ± 1.9 years of follow-up). All tests had 2.1 ± 0.3 time points, with a minimum of 2 and a maximum of 5 time points per subject per test.

### Statistical analysis

All statistical analyses were performed using R (version 4.2.1). All statistical models controlled for age at actigraphy collection, sex (31), college education (6138), baseline self-rated general health (2178), baseline body mass index (BMI; 21001), and baseline Townsend deprivation index (TDI; 189). TDI is a *z*-score transformed variable with scores lower than 0 indicating an area’s relative affluence and higher than 0 indicating relatively high material deprivation. Missing BMI, TDI, general health, and education data (a maximum of 1%) were imputed using the Amelia II package in R [30]. All data were otherwise complete.

Cox proportional hazard models were used to test associations between 24-h activity rhythm metrics with incident AD and PD using the coxphf and survival R packages. For individuals who developed AD or PD, follow-up time was calculated as the interval from actigraphy data collection to AD or PD diagnosis. Individuals were censored at the onset of AD or PD for both the progression-to-AD and the progression-to-PD analyses. For individuals who did not develop AD or PD, follow-up time was the interval from actigraphy data collection to either death or the medical record censor date of September 30, 2021.

Mann-Whitney *U* tests were used to compare fPCA scores between converters and matched controls in the AD and PD analyses.

Prospective longitudinal cognitive change was examined with linear mixed regression models using the lme4 R package. All linear mixed models included a random intercept for each participant and an interaction with time for the actigraphy measure of interest and each covariate. In addition to the covariates listed above, linear mixed models included a binary variable indicating whether the baseline assessment was in-person or remote for a given individual to account for performance differences across assessment settings. Data from linear mixed models are presented as unstandardized estimates ± standard error.

## Results

Participant demographics are summarized in Table 1. 82,829 individuals with actigraphy data were included in the study, consisting of 46,683 women (56%) and 36,146 men (44%) with a mean (± SD) age of 62.0 (± 7.8) years at the time of actigraphy data collection.

**Table 1:** Demographics at time of actigraphy collection

**Age at baseline**	62.0 ± 7.8
**Female** ***N*** **(%)**	46,683 (56)
**College or university degree** ***N*** **(%)**	35,548 (43)
**General health [1–4]**	2.0 ± 0.7
**Body mass index**	26.7 ± 4.5
**Townsend deprivation index**	− 1.7 ± 2.8
**Follow-up (years)**	6.8 ± 0.9
**Symbol Digit Substitution score**	20.4 ± 4.9
**Trail Making Test A duration (seconds)**	38.0 ± 13.7
**Trail Making Test B-A (seconds)**	25.5 ± 17.9
**Numeric Memory score**	7.04 ± 1.43
**Fluid Intelligence score**	6.68 ± 2.03
**Interdaily stability (IS)**	0.54 ± 0.12
**Intradaily variability (IV)**	0.91 ± 0.24
**Least active 5 h (L5)**	3.28 ± 6.41
**Most active 10 h (M10)**	47.4 ± 17.1
**Amplitude**	24.4 ± 9.42
**Mesor**	28.2 ± 11.7

Across all participants categorized in UK Biobank as having accelerometer data (*n* = 103,671), comparison of demographic variables between participants who were included versus excluded from our analyses revealed that participants who were excluded were younger, had lower self-reported general health, higher BMI, and higher TDI relative to participants who were included, with very small effect sizes across these differences (Cohen’s *d* ≤ 0.10).

As expected, accelerometer-derived metrics reflecting amplitude or daytime activity levels (amplitude, mesor, and M10) were highly correlated across individuals (bivariate correlation; *r* ≥ 0.70). L5 was also positively correlated with mesor (*r* = 0.70) and M10 (*r* = 0.47), but not with amplitude (*r* = 0.01). Intradaily variability and interdaily stability were negatively associated (*r* = − 0.45) suggesting individuals with more consolidated activity patterns within days (low IV) also tended to have higher regularity of activity (high IS) across days.

### Associations of 24-h rhythms with risk of AD and PD

**Fig. 2:**
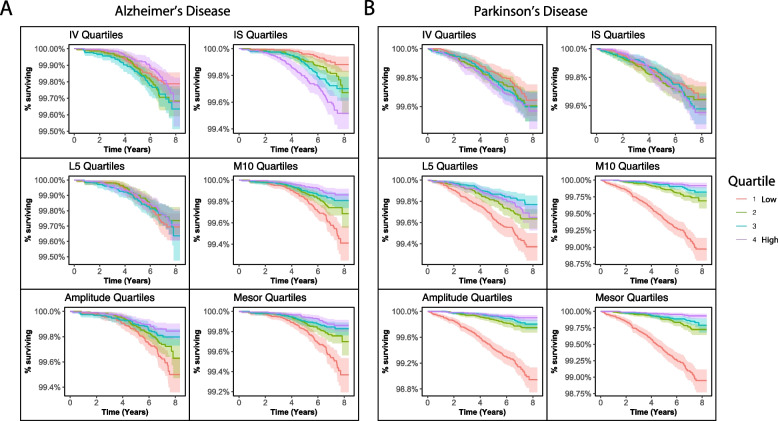
Conversion to Alzheimer’s and Parkinson’s disease by 24-h rhythm quartiles. Survival curves with 95% confidence intervals for **A** Alzheimer’s disease and **B** Parkinson’s disease based on quartiles for each of the 24-h rhythm metrics. The *y*-axes represent the percent of individuals converting to Alzheimer’s or Parkinson’s disease out of the full study sample. Abbreviations: IV, intradaily variability; IS, interdaily stability, L5, least active 5 h; M10, most active 10 h

During 8.3 years (mean follow-up 6.8 ± 0.9 years) following initial actigraphic assessment, 187 individuals converted to AD (0.2%, mean 4.8 ± 1.7 years to diagnosis) and 265 to PD (0.3%, mean 4.2 ± 1.9 years to diagnosis). Survival analyses are displayed visually in Fig. 2 and summarized in Table 2.

**Table 2:** Summary of survival analysis of 24-h rhythms and developing Alzheimer’s and Parkinson’s disease

	Alzheimer’s disease (*n* =187)			Parkinson’s disease (*n* = 265)		
	*HR*	*95% CI*	*p-value*	*HR*	*95% CI*	*p-value*
**Intradaily variability (IV)**	1.03	0.88–1.21	0.69	1.10	0.97–1.25	0.12
**Interdaily stability (IS)**	1.25	1.05–1.48	0.01	0.95	0.83–1.09	0.44
**Least active 5 h (L5)**	0.69	0.24–1.94	0.48	0.24	0.08–0.69	0.01
**Most active 10 h (M10)**	0.75	0.61–0.94	0.01	0.20	0.16–0.25	< 0.001
**Amplitude**	0.79	0.65–0.96	0.02	0.28	0.23–0.34	< 0.001
**Mesor**	0.78	0.59–0.998	0.048	0.13	0.1–0.16	< 0.001

*HR* hazard ratio: *CI*, confidence interval.

All Cox proportional hazards regression models controlled for age at actigraphy collection, sex, college education, baseline general health, baseline body mass index, and baseline Townsend deprivation index. See Fig. 2 for visualization of these data

**Fig. 3:**
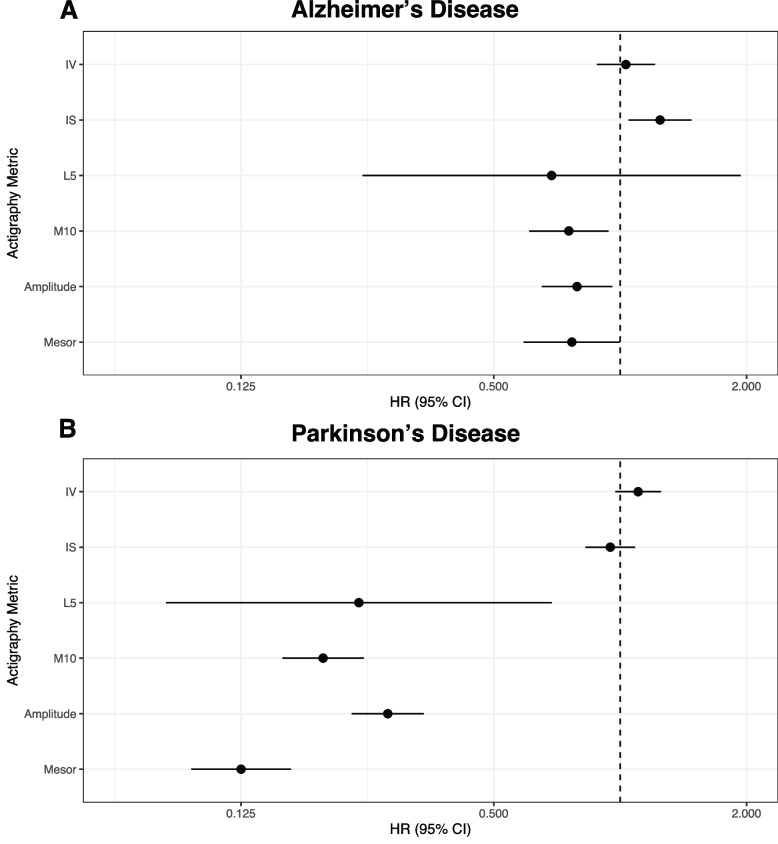
Adjusted hazard ratios for Alzheimer’s and Parkinson’s disease by 24-h rhythms. Forest plots showing standardized hazard ratios (HR) per standard deviation increase and 95% confidence intervals (CI) for developing **A** Alzheimer’s disease and **B** Parkinson’s disease, from Cox regression models including age at actigraphy collection, sex, college education, baseline general health, baseline body mass index, and baseline Townsend deprivation index. Abbreviations: IV, intradaily variability; IS, interdaily stability, L5, least active 5 h; M10, most active 10 h

An increased risk of AD was associated with higher interdaily stability, lower M10, amplitude, and mesor (Fig. 3A, Table 2) [13, 14]. An increased risk of PD was associated with lower L5, M10, amplitude, and mesor (Fig. 3B, Table 2).

### Functional principal components analysis

**Fig. 4:**
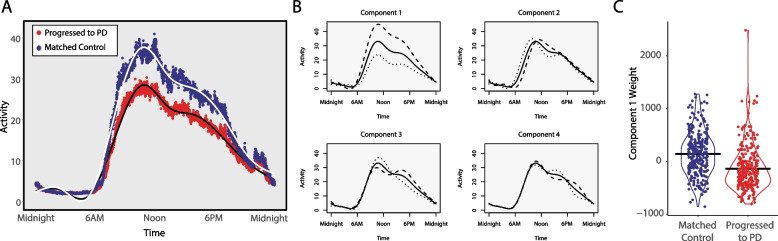
Average 24-h activity curves and functional principal components analysis for Parkinson’s disease. **A** Average activity curves in individuals who progressed to Parkinson’s disease (red) or matched individuals who did not progress to Parkinson’s disease (blue). Each plotted point represents a 30-s epoch of average activity. Daytime activity levels are visibly lower in individuals who progressed to Parkinson’s disease. **B** Four components were derived from 24-h data from individuals who progressed to Parkinson’s disease and matched controls. The solid line represents the same average activity curve in each of the four plots, plotted against clock time. Each of the four components is visualized by showing the average weight of positive fPCA scores (dashed) or negative scores (dotted) added to the average activity pattern. **C** For the first component, the right violin plot shows weights (scores) for every individual, with a black bar representing the group mean

A visualization of mean 24-h activity patterns across disease progressors and matched controls for the PD analysis is presented in Fig. 4A. The shapes and variance explained by the components were similar across AD and PD analyses, and the components for the PD analysis are presented in Fig. 4B. Component 1 could be described as an amplitude component, with higher values reflecting higher daytime activity, and explained 57% of the variance for the AD analysis and 66% for the PD analysis. Elevated component 2 represents later wake and bedtimes, with mainly flat peak activity, while lower component 2 represents earlier wake and bedtimes with a morning peak of activity; 19% of variance was captured in AD and 15% in PD analysis. Elevated component 3 represents a longer activity period interrupted by a midday dip in activity, possibly representing napping, while lower component 3 represents a flatter activity profile with slightly elevated morning activity; 12% of variance was captured in AD and 10% in PD analysis. Component 4, explaining only 6.3% for AD and 5.4% for PD, represents a late afternoon peak for those with low scores and a morning and evening peak for those with high scores.

When magnitude scores for the four fPCA components were compared between individuals who progressed to AD and matched controls, there were no differences between groups. In contrast, individuals who progressed PD showed severely reduced component 1 scores relative to individuals who did not progress to PD, suggesting lower daytime activity levels relative to matched controls (PD, − 140.4 [420.0]; controls, 140.4 [414.9]; *p* < 0.001; Fig. 4C). There were no differences for fPCA components 2, 3, or 4.

### Cognitive decline

Associations between 24-h activity metrics and cognitive decline are presented in Table 3. Worse longitudinal performance on the Symbol Digit Substitution Test was associated with lower mesor, lower M10, and lower amplitude. Increasing duration on Trails A, indicating worsening performance longitudinally, was associated with greater baseline intradaily variability, lower interdaily stability, lower M10, and lower amplitude. Increasing Trails B-A duration was associated with lower intradaily variability. Declining Numeric Memory performance was associated with greater intradaily variability. Longitudinal change in fluid intelligence score was not associated with any baseline 24-h rhythm metric.

**Table 3:** Summary of 24-h rhythm associations with longitudinal cognitive test performance

	Symbol digit (*n* = 7,533)		Trails A (*n* = 6,731)		Trails B-A (*n* = 6,629)		Numeric Memory (*n* = 7,401)		Fluid intelligence (*n* = 11,030)	
	*β (SE)*	*p-value*	*β (SE)*	*p-value*	*β (SE)*	*p-value*	*β (SE)*	*p-value*	*β (SE)*	*p-value*
IS*time	− 0.10 (0.10)	0.34	− 0.89 (0.32)	0.005	0.51 (0.51)	0.32	− 0.06 (0.04)	0.12	− 0.07 (0.04)	0.05
IV*time	− 0.005 (0.05)	0.92	0.50 (0.15)	0.001	− 0.75 (0.24)	0.001	0.05 (0.02)	0.01	− 0.01 (0.02)	0.42
L5*time	0.01 (0.01)	0.38	− 0.02 (0.03)	0.41	− 0.01 (0.04)	0.86	0.001 (0.003)	0.72	0.003 (0.003)	0.39
M10*time	0.002 (0.001)	0.01	− 0.01 (0.002)	0.03	0.001 (0.004)	0.80	− 0.0002 (0.0003)	0.59	0.0002 (0.0003)	0.51
Amplitude*time	0.002 (0.001)	0.048	− 0.01 (0.004)	0.005	0.004 (0.01)	0.52	− 0.0003 (0.0004)	0.49	0.0003 (0.0005)	0.47
Mesor*time	0.004 (0.001)	0.01	− 0.01 (0.004)	0.13	− 0.003 (0.01)	0.63	− 0.0003 (0.0005)	0.59	0.0005 (0.001)	0.36

*IS* interdaily stability: *IV* intradaily variability: *L5* least active 5 h: *M10* most active 10 h.

All linear mixed-effects models controlled for age at actigraphy collection, sex, college education, baseline general health, baseline body mass index, and baseline Townsend deprivation index. All linear mixed models included a random intercept for each participant, and an interaction with time for the actigraphy measure of interest and each covariate. Models also included a binary variable indicating whether an individual’s baseline assessment was in-person or remote

## Discussion

In 82,829 older individuals with up to 8.3 years of follow-up, we found multiple metrics of actigraphy-measured 24-h activity were associated with risk of developing Alzheimer’s disease, Parkinson’s disease, and subsequent cognitive decline. Reduced diurnal amplitude and activity levels were associated with a greater risk of both AD and PD as well as greater declines in performance on the Symbol Digit Substitution and Trails A tests. While lower regularity of rhythms across days (lower IS) was associated with declining performance on Trails A, paradoxically, higher IS was associated a greater risk of AD. Lower activity during the least active 5 h (L5) was associated with a higher risk of PD. A complementary data-driven approach (fPCA) revealed that individuals who progressed to PD had significantly lower values for a daytime activity component (which explained 66% of the variance) compared to matched individuals who did not progress to PD. Together, these data suggest objective measures of 24-h activity could be used as community-based biomarkers of neurodegeneration risk. With our rapidly growing aging population, and relatively few movement disorders and behavioral trained neurologists, non-specialists will be monitoring the majority of people at risk for neurodegenerative diseases of aging. Accelerometer-derived metrics are an affordable and scalable monitoring system that could be used by geriatricians and internists, as well as general neurologists, to evaluate elevated risk of PD, AD, and accelerated cognitive decline in all older adults.

Lower baseline activity levels (amplitude, mesor, and M10) were associated with a higher risk of both AD and PD conversion. The magnitude of these effects was greater for PD, with the majority of individuals who converted to PD being in the lowest quartile for amplitude, mesor, and M10. The association between accelerometer-derived activity and PD progression was described in another recent UK Biobank study [31] which utilized a measure of average acceleration rather than the rest-activity rhythm metrics reported here. Our results further align with previous reports in smaller cohorts demonstrating links between reduced activity and diurnal amplitude and risk of dementia and cognitive impairment [13, 14] and PD [15]. Moreover, the matched fPCA analysis, which was naïve to the shape of 24-h activity, revealed a daytime activity component that was associated with PD progression, in agreement with the cosine-based analyses. This fPCA result suggests that daytime activity may be the most robust disease-specific difference in 24-h activity that specifically determines those at risk for PD. Indeed, the fPCA in AD converters did not reveal differences in any of the 24-h components, suggesting there was no consistent 24-h activity “signature” that differed between those who converted to AD and matched non-converters.

Beyond activity and amplitude, greater regularity of rhythms across days (IS) was associated with a higher risk of conversion to AD. While this finding goes against a framework wherein circadian rhythms weaken in preclinical AD and are responsible in part for rhythm regularity [6, 32], it may reflect IS values being driven by factors other than circadian biology—for example, aging individuals with monotonous routines could be at increased risk for AD. Two previous studies found associations between lower daily rhythm regularity and subsequent risk of cognitive decline [11, 14]. The cohorts in these studies had a mean age at baseline of 76 and 82, compared to mean age 62 in the present UK Biobank study. Thus, our findings do not necessarily contradict these previous reports—it is possible that rhythm regularity may be sensitive to different aspects of lifestyle and behavior at different points in adulthood.

Multiple mechanisms may contribute to the link between reduced 24-h rhythms and risk of incident PD. Brainstem circuits involved in sleep-wake regulation are affected early in the disease process, before the onset of dopaminergic loss [4, 32, 33]. Relatedly, daytime napping [34] and excessive daytime sleepiness [35, 36] are both associated with elevated risk of PD. Another explanation is that the presence of subclinical rigidity and bradykinesia in the prodromal phase of the disease may constrain movement, resulting in lower overall activity quantification [37]. This explanation is supported by our finding that PD risk was associated with lower L5 values, a proxy for nocturnal activity levels. Low L5 values in these individuals, rather than reflecting consolidated and restful sleep, are likely capturing nocturnal rigidity in prodromal disease stages. Importantly, low L5 was the only marker in our analysis specific in predicting subsequent PD diagnosis. REM sleep behavior disorder (RBD), the loss of skeletal muscle atonia typically occurring during REM sleep, is present in 16–47% of individuals with PD and frequently experienced years before a PD diagnosis [38]. Recent work has shown that RBD-related movement can be detected with actigraphy devices [39, 40]; however, it is not clear how lack of atonia during REM sleep would impact L5. The markedly higher effect sizes for actigraphy metrics predicting PD progression, relative to AD progression, may be due to higher prevalence of sleep disturbance in PD (estimated at up to 80% [41],) as well as PD-related rigidity affecting accelerometer measures. Future studies with more detailed measures of sleep (including RBD), motor impairment, and disease biomarkers could untangle the relative contributions of rigidity, RBD, and impaired sleep-wake regulation to the observed associations.

The links between impaired 24-h rhythms and subsequent cognitive decline are consistent with previous reports in smaller longitudinal samples of older adults, which have similarly reported lower mesor and amplitude predicting cognitive decline in women [10] and in men [12]. In the present study, baseline amplitude, mesor, and M10 predicted worse longitudinal performance on the Symbol Digit Substitution Test, an assessment of processing speed and executive function [42]. Performance on Trails A, the numeric portion of the Trail Making Test and considered a measure of processing speed [43], also showed greater decrease over time in individuals with lower baseline amplitude and M10. Higher amplitude, mesor, and M10 were also associated with a lower risk of developing AD, confirming previous reports in smaller studies of actigraphy metrics and AD progression. Together, these associations agree with literature suggesting higher activity levels may contribute to preserved cognitive function [8, 44] or conversely that attenuated physical activity may serve as an indicator of looming decline.

The present associations between cognition and nonparametric measures of 24-h rhythms offer a less straightforward interpretation. On one hand, declining performance on Trails A was associated with greater within-day rhythm fragmentation (IV) and weaker across-day rhythm stability (IS) at baseline. This finding is in line with hypothesized relationships between circadian rhythm robustness and cognitive aging [32, 45] as well as a recent report that high IV and low IS are associated with risk of cognitive impairment [11]. Conversely, we found higher IV was associated with *better* longitudinal performance on Trails B-A (a measure of executive task-switching) and Numeric Memory (a test of working memory), suggesting that individuals with more fragmented rhythms at baseline had less decline in these domains. These relationships raise the possibility that despite including multiple demographic and lifestyle covariates in our analyses, some cohort-specific effect may be driving IV values such that higher “fragmentation” of 24-h activity actually reflects better brain health in at least a proportion of individuals.

### Limitations

This study has some limitations. First, the study did not include biological markers of disease or genetic information that could elucidate whether some individuals were at prodromal disease stages at the time of actigraphy collection. Second, we did not have objective sleep measures. Actigraphy lacks specificity in differentiating sedentary activity from nocturnal sleep and daytime napping [46]. Given the absence of concurrent sleep logs or sleep timing information in the UK Biobank actigraphy data, we elected to take an agnostic approach by characterizing 24-h sleep-wake rhythms rather than purporting to detect sleep based on activity. Third, our study relied on ICD-10 codes and self-reported diagnoses to determine AD and PD, without a specification for dementia with Lewy bodies or PD dementia, which could have led to underestimation of associations based on disease misclassifications. The incidence of AD and PD diagnosis in the UK Biobank may differ from incidence in the general population, and potential biases such as attrition due to cognitive impairment [47] or prescribed treatment of motor symptoms may contribute to these biases. The age range of the UK Biobank cohort, and differences in the average age of onset between AD and PD, may have also contributed to the relative incidence of both diagnoses during the follow-up period. Finally, the modest effect sizes of associations with actigraphy metrics, particularly in predicting progression to AD, limit the clinical utility of our findings. Future studies should utilize deeply phenotyped cohorts with biomarkers of AD and PD in order to determine what stage of pathological progression may be best suited for actigraphy-based detection of 24-h rhythm impairment.

## Conclusions

The present results suggest that 24-h rhythm integrity as assessed by seven days of wrist actigraphy can serve as a prospective marker of incident AD and PD risk as well as cognitive decline.

## Data Availability

All data are available from the UK Biobank study (https://www.ukbiobank.ac.uk).

## Abbreviations

AD: Alzheimer’s disease
PD: Parkinson’s disease
IV: Intradaily variability
IS: Interdaily stability
L5: Least active 5 h
M10: Most active 10 h
HR: Hazard ratio
CI: Confidence interval
fPCA: Functional principal components analysis
